# Surface Modification of Bioactive Glass Promotes Cell Attachment and Spreading

**DOI:** 10.1021/acsomega.1c02669

**Published:** 2021-08-25

**Authors:** Latifeh Azizi, Paula Turkki, Ngoc Huynh, Jonathan M. Massera, Vesa P. Hytönen

**Affiliations:** † BioMediTech, Faculty of Medicine and Health Technology, Tampere University, Kauppi Campus, Arvo Ylpön katu 34, 33520 Tampere, Finland; ‡ Laboratory of Biomaterials and Tissue Engineering, Faculty of Medicine and Health Technology, Tampere University, Hervanta Campus, Korkeakoulunkatu 3, 33720 Tampere, Finland; § Fimlab Laboratories, Biokatu 4, 33520 Tampere, Finland

## Abstract

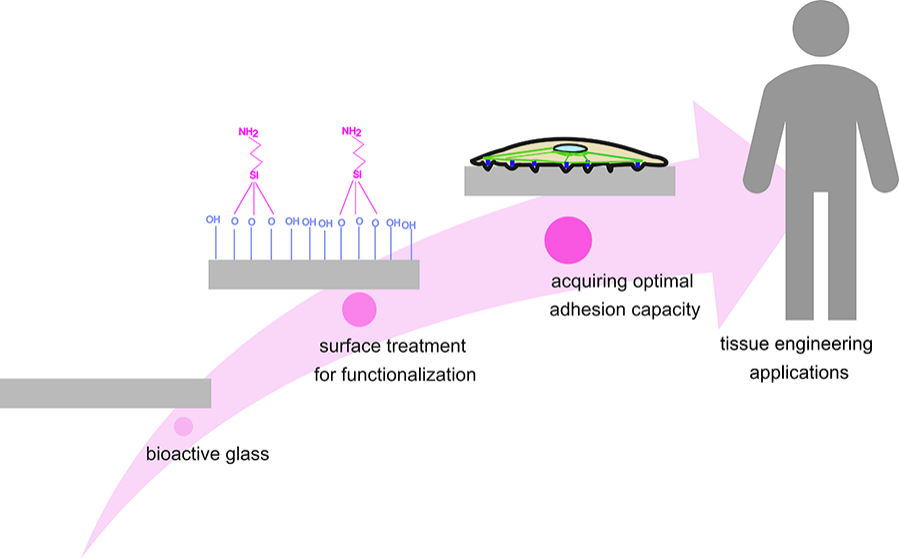

Phosphate glasses have several advantages over traditional silicate-based bioglasses but are inferior in the crucial step of cell attachment to their surface. Here, as a proof of concept, we analyze fibroblast attachment to the phosphate glass surface subjected to basic treatment and silanization. Silicate (S53P4)- and phosphate (Sr50)-based bioactive glasses were either untreated or surface-treated with basic buffer and functionalized with silane. The surface-treated samples were studied as such and after fibronectin was adsorbed on to their surface. With both glass types, surface treatment enhanced fibroblast adhesion and spreading in comparison to the untreated glass. The surface-treated Sr50 glass allowed for cell adhesion, proliferation, and spreading to a similar extent as seen with S53P4 and borosilicate control glasses. Here, we show that surface treatment of bioactive glass can be used to attract cell adhesion factors found in the serum and promote cell–material adhesion, both important for efficient tissue integration.

## 1 Introduction

Cell adhesion, proliferation, and communication with the extracellular matrix (ECM) can be manipulated by the composition and physical properties of the cell culturing substrate (including surface stiffness, porosity, chemistry, and charge). In the development of biomedical products, providing maximal patient safety is a great challenge. Therefore, safe and good-quality biomaterials are critical factors, e.g., for successful implant integration. When a biomaterial is deployed into a patient, it faces a complex biological environment with different proteins (such as fibronectin and fibrinogen), which can act as ligands for receptors such as integrins to support cell attachment. The physicochemical surface properties play a major role in the cell adhesion process. Therefore, the ability of the surface to attract these biological adherence factors is a key step in optimization of performance of bioactive materials.^1−3^

Bioactive glasses have been widely studied, and among them, silica-based bioactive glasses are commonly used in various clinical applications such as dental and orthopedic applications.^4^ Despite several good qualities, unsuitability for hot-processing and the lack of mechanical strength are the main drawbacks for several applications.^5^ Another drawback of silica-based glasses is their slow degradation or even lack of degradation in some cases. For example, remnants of silica glass have been found even 14 years after their implantation.^6,7^

Phosphate glasses (PGs) have been studied in the past for their ability to degrade in a congruent manner, providing a more complete dissolution of the material over time in comparison to the silicate-based glasses. Their degradation can be adjusted by modifying their composition. PGs can be drawn into fibers^8^ and sintered into scaffolds due to their wide thermal stability.^9^ Another advantage of PGs is that they can be easily doped with different metal ions with therapeutic interest.^10−13^ For example, phosphate-based bioactive glasses have been doped with silver,^14^ copper, and iron to influence their thermal stability, dissolution kinetics, structure, and antimicrobial properties.^15^ Strontium-containing phosphate-based Sr50 used here was studied for biocompatibility using human gingival fibroblasts. It was shown that the released strontium ions could, after 7 days, enhance cell proliferation. However, for the first 3 days, the cells were struggling to attach to the glass surface, and the cell count decreased from day 1 to day 3,^16^ suggesting that the initial cell attachment and proliferation should be enhanced to promote the biomedical use of the material.

Cell adhesion on biomaterial surfaces is important for many tissue-engineering applications. In general, in tissue engineering, the first criterion for the scaffold surface is that it should permit cell attachment and adhesion. In previous studies, cell adhesion has been facilitated by modification of physical properties of the material, e.g., increasing the surface roughness at the nanometer scale or by chemical components, e.g., grafting of adhesion peptides such as integrin ligand RGD (Arg-Gly-Asp). 3-Aminopropyltriethoxysilane (APTES) has been known as a suitable organosilane and intermediator for further functionalization of biomaterials.^17^ Specifically, the presence of a reactive functional group such as hydroxyl group (OH)^18^ and the optimization of the APTES grafting mechanism (referred to as silanization in this study) have been studied for silicate-based^19,20^ and phosphate-based^21^ bioactive glasses. In all cases, the successful grafting of APTES was studied using X-ray photoelectron spectroscopy, with specific focus on the Si, O, and N high-resolution spectra.

**Figure 1:**
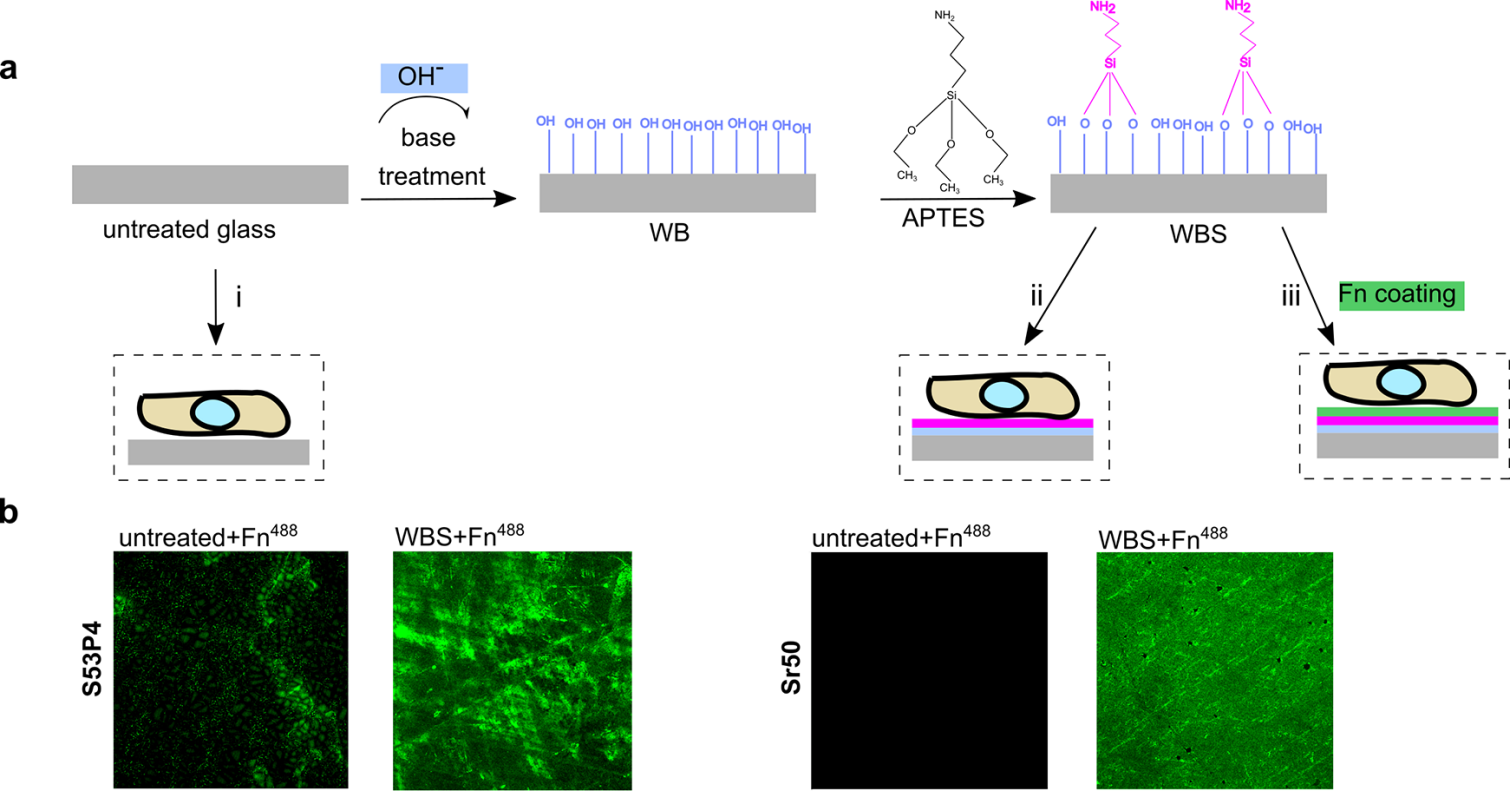
Bioactive glass functionalization to enhance cell compatibility. (a) Schematic representation of the experimental procedure. The untreated glass is first immersed in a basic buffer, followed by silanization and fibronectin grafting. The biocompatibility of the materials was assessed by culturing the cells on the untreated glass (i), treated glass (ii), and Fn-grafted glass (iii). (b) Confocal fluorescence microscopy images of the untreated and base-wash + silane-treated glass surfaces (S53P4 and Sr50) coated with 10 μg/mL Alexa-488 fluorescently labeled fibronectin (green fluorescent). The base-wash treatment followed by silanization significantly improved fibronectin grafting on the surface. The fluorescence microscopy images in panel (b) are reprinted with permission from ref (34). Copyright 2021 American Chemical Society.

Lately, in the work by Huynh et al.,^34^ we aimed toward enhancing the protein absorption by surface treatment of the glass surface (Figure 1a). We found that PG treated with basic wash (10 mM Tris-HCl pH 9) enhances surface hydroxylation with free −OH groups, improving the successful amino-silanization and further fibronectin grafting.^34^ Enhancement of the grafting of fluorescently labeled fibronectin was demonstrated using confocal fluorescence microscopy (Figure 1b).

In this study, we wanted to determine if the surface treatment would allow efficient cell adhesion of mouse fibroblasts on the phosphate-based Sr50 glasses. Cell behavior and morphology were compared to those of cells cultured on top of silicate-based S53P4 and cell culture-compatible borosilicate coverslips. We tested the effect of the base-wash treatment on cell spreading, movement, and morphology as well as the composition of adhesion proteins such as paxillin involved in cell–material communication.^22^

## 2 Results and Discussion

### 2.1 Cell Adherence and Movement are Influenced by Surface Treatment

Phosphate-based glasses are based on phosphorus pentoxide (P_2_O_5_), which is also the glass network former, as silicate (Si) in the silicate-based glasses. From the biomedical application point of view, phosphate-based glasses have unique properties. For example, they dissolve completely in an aqueous solution, and their overall dissolution rate can be controlled. The possibility of developing a phosphate-based glass that is tough and shows high resistance to fracture makes phosphate-based bioglasses attractive alternatives to silicate-based glasses.^8^

**Figure 2:**
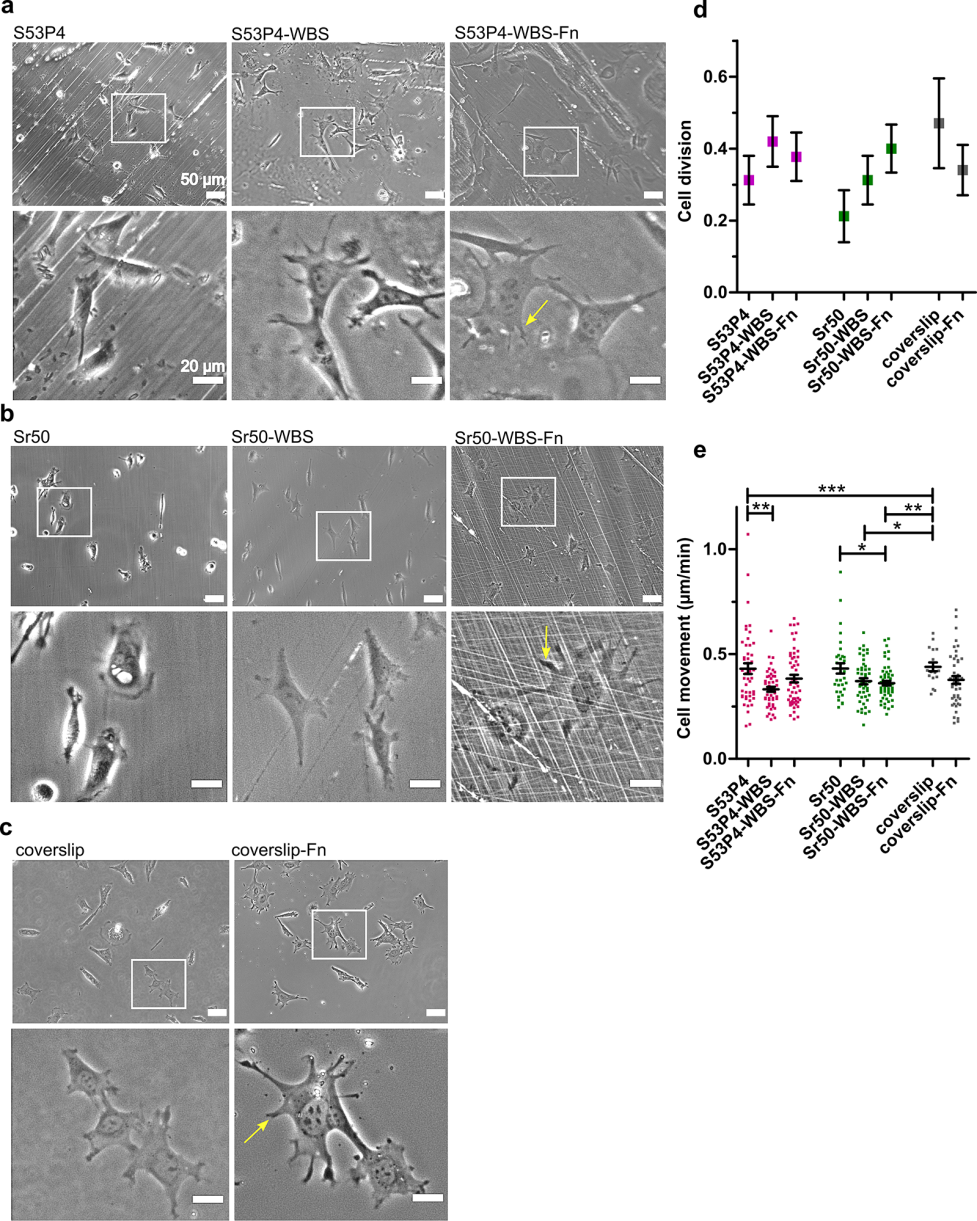
Treatment of bioactive glass influences cell adhesion, cell proliferation, and the migration rate. (a–c) Snapshots from time-lapse videos from the first hour of imaging (20× objective). (a) Cells on silicate-based S53P4 glass (untreated, WBS-treated, and WBS-Fn-treated) (top panel) and magnified images (bottom panel). (b) Cells cultured on phosphate-based Sr50 glass (untreated, WBS-treated, and WBS-Fn-treated (top panel) and magnified images (bottom panel). (c) Cells cultured on glass coverslips (without and with fibronectin coating (top panel) and magnified images (bottom panel). (d and e) Average cell division, *N* ∼ 45 cells, for each condition (d) and the cell movement (μm/min), *N* ∼ 45 cells, for each condition (e) for each type of glass in 12 h. The statistical significance in (d) and (e) was analyzed by the *t*-test and the Mann–Whitney test: *P < 0.05, **P < 0.01, and ***P < 0.001; bars represent the mean values with SEM. The experiments were repeated at least three times on separate occasions. In the magnified panel at the bottom of each section, adhesion on the tip of the membrane extension is shown with a yellow arrow. The scale bar in the upper images in each set is equal to 50 μm and in the magnified lower images, 20 μm.

To evaluate the impact of surface treatment on cell adhesion, cell movement, and proliferation, cells were monitored using time-lapse imaging for 12 h. For each glass type, we used three conditions: (1) untreated material, (2) surface-treated material (washed with a basic solution and silanized; WBS), and (3) surface-treated material precoated with fibronectin (WBS-Fn). Figure 2 shows snapshots of the cells growing on the glasses’ surface (Figure 2a,b). Borosilicate glass coverslips with and without fibronectin coating were used as the positive control (Figure 2c). A previous study has shown that CCD-18CO fibroblasts attach on the surface of S53P4 glass without any changes in cell behavior.^23^ Here, the same phenomenon was observed for the fibroblasts cultured on untreated S53P4 glass with an elongated cell shape. However, the average cell surface area was smaller and the cells appeared less adherent (as indicated by halo artifacts next to the cell boundaries) in comparison to the cells cultured on top of the borosilicate control glass or on surface-treated or surface-treated and Fn-grafted S53P4 (Figure 2a). With Sr50 glass, the cells appeared even smaller with a less elongated morphology and poor adherence on the untreated glass surface. Again, surface treatment-induced cell adherence and Fn-grafting was able even to further increase the cell adherence as seen with larger adhesion sites on the cell extensions (Figure 2b, arrows). These results indicated that surface treatment was able to improve the cell compatibility of Sr50 and S53P4 glasses (Supporting Videos 1–6).

Cell movement on the surface and their proliferation were quantified from time-lapse videos of differentially treated materials altogether from three independent experiments (Figure 2d,e). To evaluate the influence of different glasses on cell proliferative activity, cell division was tracked during 12 h of cultivation. We found that surface treatment had a negligible effect on cell proliferation compared to the untreated samples (Figure 2d). Commonly, better surface adhesion enables cell movement but can also slow it down due to increased adhesion. Surface treatment of S53P4 significantly decreased cell movement, suggesting that the treatment alone could be able to improve cell adhesion. Fn-grafting had a negligible effect on cell movement (Figure 2e). As the cell culture media used here contain serum, untreated and treated glasses become grafted with the protein components of the serum, including fibronectin. Surface-treated Fn-grafted Sr50 showed decreased cell movement, possibly indicating enhanced cell adhesion (Figure 2e).

### 2.2 Cell Adhesion to Bioactive Glass can be Improved Further by Fibronectin Grafting of the Treated Surface

**Figure 3:**
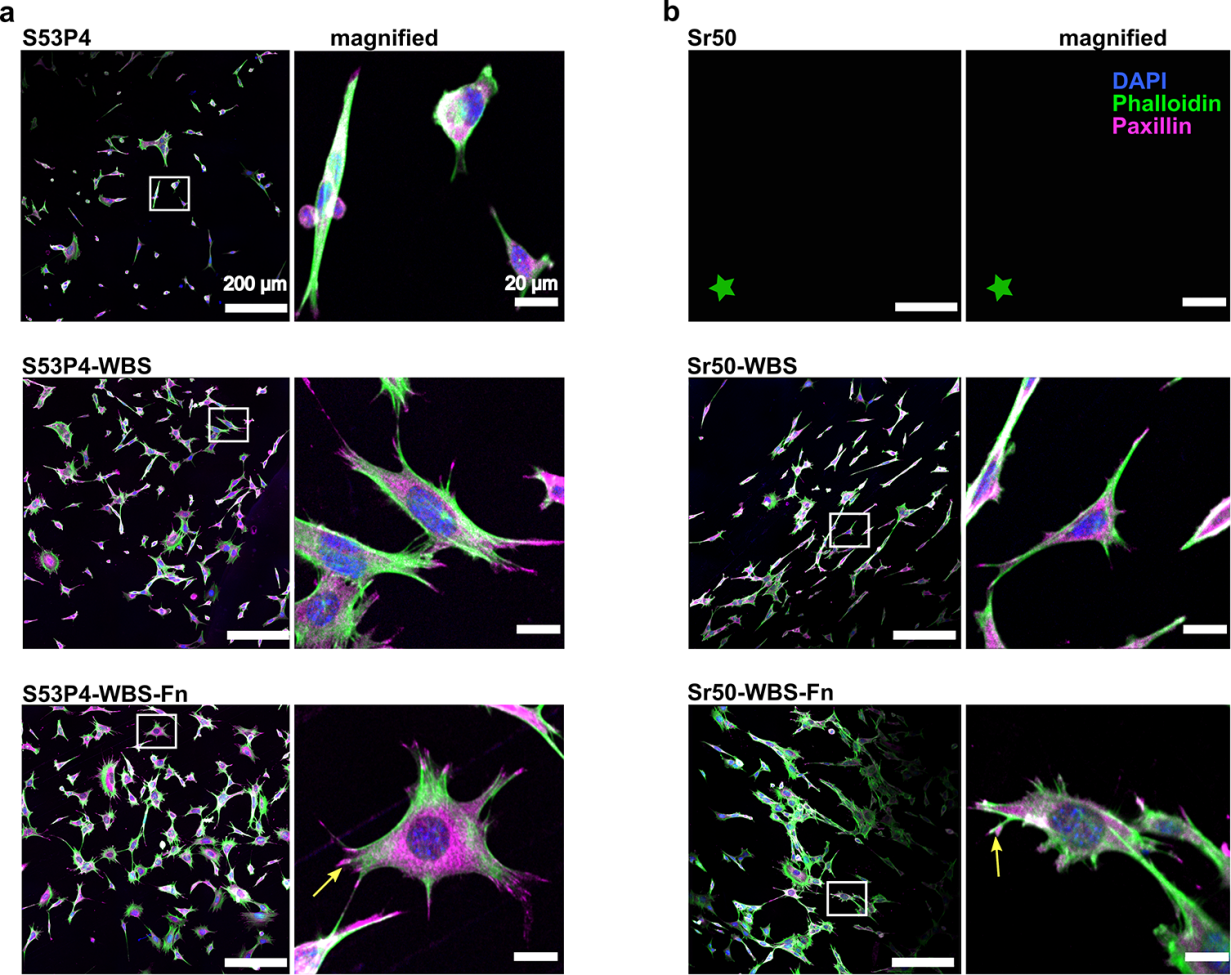
Silane treatment and fibronectin grafting enhance cell adhesion. (a and b) Confocal microscopy images of the cells cultured on WBS-treated and WBS-Fn-treated samples (WBS = bioglass washed in basic buffer and silanized and WBS-Fn = bioglass washed in basic buffer and silanized and fibronectin-coated) (left panels, scale bar is 200 μm). Magnified images (right panels) are with a scale bar of 20 μm. The green star in (b) indicates that data is not available since the cells were washed out from the Sr50 untreated glass during the immunostaining process. DAPI staining is shown in blue, phalloidin (actin cytoskeleton) in green, and paxillin in magenta. In the magnified panel, the mature adhesion plaque on the tip of the membrane extension is indicated with a yellow arrow.

**Figure 4:**
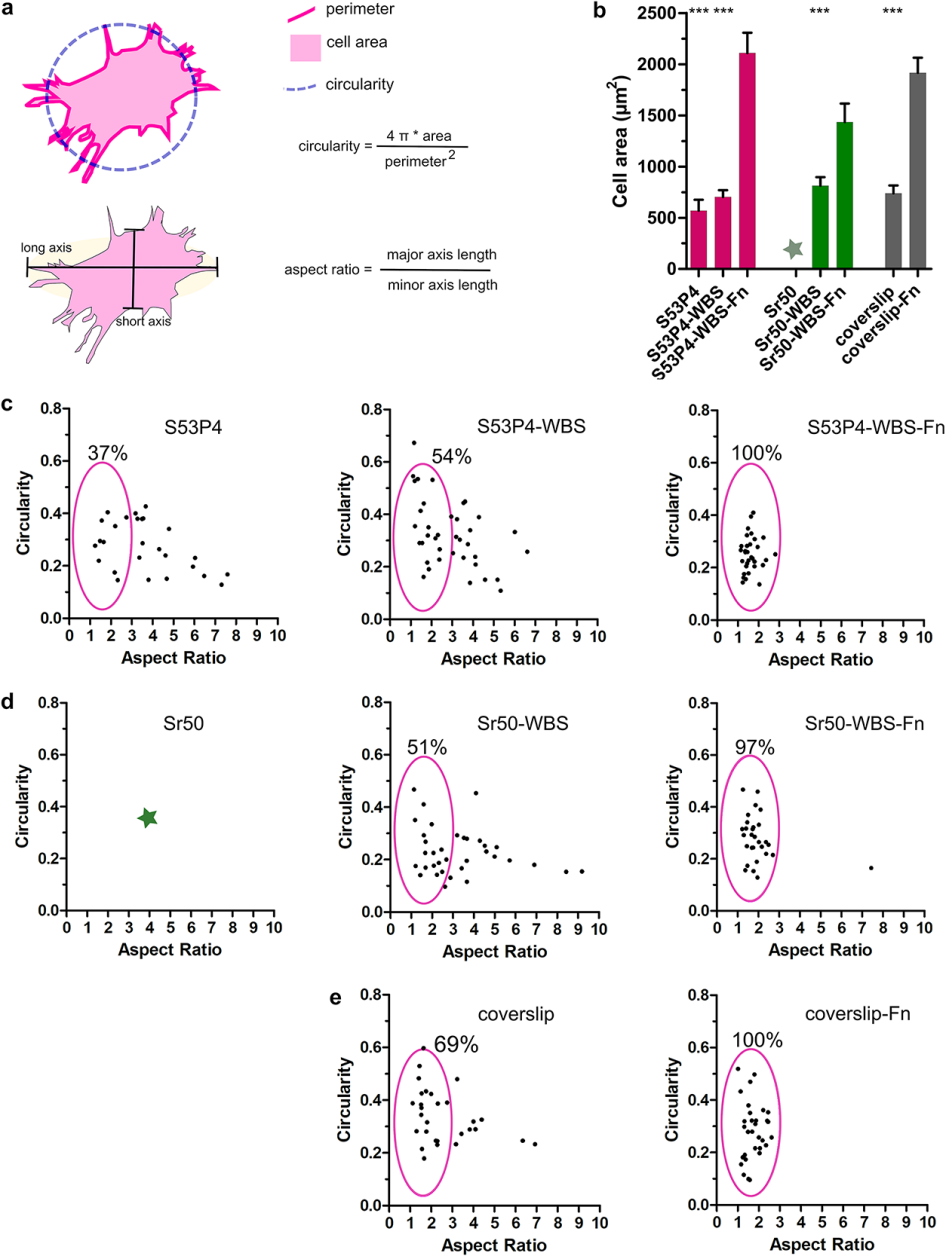
Morphological phenotype of cells cultured on various substrates. (a) Schematic illustration of the morphological parameters; the area covered by the cell is known as “cell area”, the ratio of the cell’s longest length and the shortest width is called the “aspect ratio (AR)”, the distance around the cell is called “perimeter”, and the normalized ratio of cell area and the perimeter is called “circularity”. (b) Cell area measured for fibroblasts cultured on various materials. *N* ∼ 30 randomly selected cells analyzed for each sample. The statistical significance was analyzed by the paired *t*-test: *P < 0.05, **P < 0.01, and ***P < 0.001; bars represent the mean values with SEM. (c–e) Plot of circularity vs AR for each type of glass. Each data set represents >30 randomly selected cells. The green star in (b) and (d) indicates that data is not available since the weakly adhered cells were washed out from the Sr50 untreated glass during the sample processing.

Surface modification by chemical treatment is a simple way to modify the surface morphologies^24^ and improve cell adhesion.^25^ To understand the effects caused by different surface treatments (change in surface charge and chemical structure)^34^ on cell adhesion signaling, we analyzed the behavior of the cells in more detail (Figures 3 and 4) using immunostaining and confocal fluorescence microscopy. We targeted paxillin in immunostaining since this protein is a known marker for focal adhesion sites that control cell–substrate interaction.^26^ Cells on the top of untreated S53P4 did not contain notable adhesions, and paxillin was mainly found scattered in the cytoplasm. Cells cultured on top of untreated Sr50 were lost during the immunostaining procedure, indicating significant defects in adhesion to the surface. With both glass types, cells cultured on top of the surface-treated glasses showed paxillin-rich adhesion sites on the tips of their protrusions. However, the cell morphology seemed elongated, and the adhesions were low in number. When cells were cultured on top of the surface-treated and Fn-grafted glasses, paxillin-rich adhesions were larger in size (Figure 3a,b; arrows) and the cell morphology was more symmetrical (Figure 3a,b).

In addition to the visual analysis of focal adhesion sites, we also quantified the changes in cell morphology when cultured on different substrates. The general features for cell morphology were assessed using an image analysis tool as described in the Materials and Methods section and schematically in Figure 4a. Quantification of the cell area revealed that cells cultured on the surface-treated S53P4 glass were slightly larger (700 ± 66 μm^2^ compared to cells grown on the untreated (570 ± 105 μm^2^ glasses. However, with Fn-grafting on top of the treated glass, the cell area increased significantly (2100 ± 197 μm^2^, suggesting that the treatment could aid in Fn-grafting of the S53P4 glass. In the case of Sr50 glass, the effect was undeniable; without treatment, cells were lost during the labeling procedure most likely due to poor adhesion, whereas with the surface treatment, we saw large, adherent cells with a cell area of 813±84 μm^2^. When treated Sr50 glasses were surface-treated and preincubated with Fn prior to cell culture, we could increase the cell size even further to 1435 ± 180 μm^2^. Treated Sr50 glass resulted in cell spreading comparable to the cell culture-compatible borosilicate coverslip (mean cell area of 738 ± 79 μm^2^, and Fn-coated surface-treated Sr50 glass resulted in cell spreading comparable to the Fn-coated coverslip (mean cell area of ∼1916 ± 147 μm^2^ (Figure 4b).

These findings further verified the poor cell culture compatibility of the untreated Sr50 glass and showed that cell adhesion and spreading can be improved by the surface treatment of both S53P4 and Sr50 glasses.

Using cells cultivated on Fn-treated coverslip as a reference, the measured data was “gated” to evaluate the characteristics of the cell population. For the cells cultivated on untreated S53P4 glass, only 37% of the cell population was found in the gated area with the rest having higher AR values, suggesting a more elongated morphology (Figure 4c). With WBS treatment, 54% (S53P4-WBS) and 51% (Sr50-WBS) of the cell population were within the gated area and the scatter plot resembled the one seen with the borosilicate coverslip (69%) (Figure 4c–e). Fibronectin grafting on top of WBS-treated glasses further changed the cell morphology with 100% and 97% of cells within the gated area for S53P4-WBS-Fn and Sr50-WBS-Fn glasses, respectively, resembling the scatter plot of the Fn-coated borosilicate coverslip (100%) (Figure 4c–e).

Nevertheless, in surface-treated glasses with Fn grafting, the cell area and morphology changed similarly as seen with cells cultured on top of Fn-coated borosilicate coverslips, suggesting that with this simple method we can create similar adhesive properties of the bioactive glass to those of commonly used borosilicate coverslip used in cell culture laboratories. In addition, these results show that surface treatment alone is sufficient to attract the adhesion factors found in the serum and to promote cell growth.

## 3 Conclusions

Phosphate-based bioactive glasses have a unique set of properties such as the controllable dissolving rate with the release of ions during the degradation process to promote cell growth. However, the initial cell adhesion to these glass surfaces is poor. Here, we showed that with simple surface treatment (base-washing, silanization, and Fn-grafting) of the Sr50 glass, we can promote cell adhesion and spreading to a similar extent as with materials commonly used in cell culture. In addition, our results suggest that surface treatment could also induce serum- and Fn-grafting of S53P4, the commonly used silicate-based bioactive glass.

Altogether, these results indicate that phosphate bioactive glasses can be a promising substitute for traditional silicate bioactive glasses for applications in tissue engineering.

## 4 Materials and Methods

### 4.1 Preparation of Different Glass Types

Chemical compositions of various bioactive glasses are shown in Table 1. The surface treatment, washing steps, and silanization of the glasses were performed as explained in ref (34).

**Table 1:** Composition of the Silicate-Based S53P4^27^ and Phosphate-Based Sr50^28^ Glasses (mol %)

	SiO_2_	Na_2_O	P_2_O_5_	CaO	SrO
S53P4	53.85	22.66	1.72	21.77	
Sr50		10	50	20	20

Shortly, after preparing the glass discs, they were polished mechanically and washed by immersing them in a basic buffer solution (10 mM Tris-HCl, pH 9) for 6 h at room temperature (RT). They were then dried and silanized using 1 mmol/L APTES (Alfa Aesar) dissolved in ethanol for 6 h at RT.^21^ Samples were dried at 100 °C for 6 h to strengthen the bonding between silane and glass. Excess APTES was removed by sonicating them three times in ethanol, followed by drying at 100 °C for 1 h. The silanized samples were stored in a desiccator. We utilized silicate-based glass (S53P4) and phosphate-based glass (Sr50) in our experiments. For each glass type, we used three conditions: (1) untreated material, (2) surface-treated material (washed with a basic solution and silanized; WBS), and (3) surface-treated material precoated with fibronectin (WBS-Fn).

### 4.2 Fibronectin (Fn) Coating

Previously,^34^ we found that the treatment of bioactive glasses with basic buffer is a preferential condition for fibronectin grafting (Figure 1b). A part of the sample was fibronectin-coated (Fn-coated) before the cell culture experiment by treating the bioactive glass sample with 10 μg/mL fibronectin in PBS (69 mM NaCl, 1.3 mM KCl, 19.6 mM Na_2_HPO_4_·2H_2_O, 3.3 mM KH_2_PO_4_, pH 7.4) for 1 h at RT. Fibronectin was purified from human plasma (Octaplas) using gelatin affinity chromatography (Gelatin-Sepharose 4B; GE Healthcare) following the principles described by Ruoslahti et al.^22^ After elution, fibronectin was dialyzed in PBS and the purity was confirmed with SDS-PAGE, followed by storage at −20 °C. The biological activity of the affinity-purified fibronectin has been confirmed previously.^29,30^

The grafting of fibronectin on different glasses was quantified using fluorescently labeled fibronectin as described in detail in ref (34). The Fn-grafted glasses were kept in the dark before imaging using Nikon A1R (+laser scanning with an A1-DUG GaAsP Multi Detector Unit, Tokyo, Japan), 20×/0.75, Nikon Plan Apo VC air objective.

### 4.3 Time-Lapse Imaging and Immunostaining for Confocal Imaging

For the cell experiments, mouse embryonic fibroblast (MEF) cells (a gift from Dr. Wolfgang Ziegler; described by Xu et al.^31^ were maintained in high-glucose Dulbecco’s modified Eagle’s medium supplemented with 10% fetal bovine serum and 1% penicillin/streptomycin in a humidified 37 °C, 5% CO_2_ incubator. Surface-treated bioactive glasses and untreated control samples were fixed to the bottom of a 12-well plate (MatTek Corporation, USA, containing a cover glass of 14 mm diameter) using polystyrene (PS) liquid glue (made by dissolving rigid PS in xylene). A normal borosilicate glass coverslip was used as a control (VWR, diameter of 13 mm, thickness of 0.16–0.19 mm). Surface-treated and fibronectin-grafted (WBS-Fn) glasses were obtained with 1 h of incubation at RT with 10 μg/mL fibronectin (in PBS). Plain surface-treated glasses were kept in PBS for 1 h at RT.

MEF cells were detached from the cell culture flask using trypsin treatment (Gibco, TrypLE Select 1×, REF#12 563 011), and they were allowed to attach on various bioactive/control glass samples in a +37 °C cell culture incubator for 2 h before time-lapse imaging with an EVOS FL Auto microscope (Thermo Fisher Scientific) for 12 h. After time-lapse imaging, the cells were fixed using 4% paraformaldehyde (PFA) for 20 min and washed gently with PBS. The cells were permeabilized and immunostained using the standard protocol. Briefly, to visualize the cell-ECM adhesion sites, cells were labeled with antipaxillin (1:200, BD laboratories #610 051) for 1 h at RT, followed by 3 × 5 min washes with PBS. Secondary antibody (goat antimouse, AlexaFluor 568, 1:250, Molecular Probes #A11004) was used to detect the paxillin antibodies. Actin filaments were stained using fluorochrome-conjugated phalloidin (Phalloidin-AlexaFluor-488, 1:40, Life Technologies). Cells were mounted using ProLong Gold Antifade Mounting (Invitrogen, Thermo Fisher Scientific) containing 4′,6-diamidino-2-phenylindole (DAPI) to stain the nuclei.

Immunostained samples were imaged using a Nikon A1R+ laser scanning confocal microscope (Plan Apo VC 20× DIC N2, N.A. 0.75, WD 1.00 mm air).

### 4.4 Migration Speed, Proliferation, Cell Area, and Morphology Analysis

The time-lapse images captured with an EVOS FL Auto microscope (20× objective) were analyzed manually using ImageJ (Fiji).^32^ The MTrackJ plugin^33^ was used to analyze the cell migration rate. Cell division (proliferation) was tracked manually from the same cells investigated in the migration speed analysis by calculating the number of times the cell divided within 12 h.

Cell area and shape analyses were performed using the ImageJ free-hand selection tool by manually drawing the area of the cell from the confocal microscope images of fixed cells and using the “measurement analysis” tool in the software. The shape analyses of the MEF cells, AR, and circularity determination were performed in the same way using ImageJ software.

The mathematical formula used to calculate 2D morphometric descriptions for cell shape analysis, which is calculated automatically by ImageJ (Fiji) for circularity, is 4pi × (area/perimeter^2^). A value of 1 indicates a perfect circle. The formula for calculating the AR is major axis length/minor axis length.^32^

### 4.5 Statistical Analysis

Three independent experiments were carried out for each set of analysis. The statistical significance between the samples in the cell movement and division analysis (*N* ∼ 45 cells for each sample) was studied using the *t*-test and the Mann–Whitney test: *P < 0.05, **P < 0.01, and ***P < 0.001 and bars represent the mean values with SEM. The statistical significance for the randomly selected cells (*N* ∼ 30) for analyzing the cell area and plotting the circularity vs AR in each type of glass was analyzed by the paired *t*-test: *P < 0.05, **P < 0.01, and ***P < 0.001 and bars represent the mean values with SEM.

## Supporting Information Available

Time-lapse videos 1–6 obtained from an EVOS FL Auto microscope for analyzing movement of cells cultured on top of the bioactive glass (S53P4, S53P4-WBS, S53P4-WBS-Fn, Sr50, Sr50-WBS, Sr50-WBS-Fn) for 12 h (AVI, AVI, AVI, AVI, AVI, AVI)

## Supplementary Material

ao1c02669_si_001.avi

ao1c02669_si_002.avi

ao1c02669_si_003.avi

ao1c02669_si_004.avi

ao1c02669_si_005.avi

ao1c02669_si_006.avi

## Abbreviations

PG: phosphate glass
APTES: aminopropyltriethoxysilane
RT: room temperature
WBS: washed with a basic solution and silanized
Fn: fibronectin
MEF: mouse embryonic fibroblasts
AR: aspect ratio.
